# Impact of specific immunotherapy and sting challenge on the quality of life in patients with hymenoptera venom allergy

**DOI:** 10.1016/j.waojou.2021.100536

**Published:** 2021-04-21

**Authors:** Tamara Eitel, Kim Nikola Zeiner, Katharina Assmus, Hanns Ackermann, Nadja Zoeller, Markus Meissner, Roland Kaufmann, Stefan Kippenberger, Eva Maria Valesky

**Affiliations:** a Department of Dermatology, Venereology and Allergology, Goethe University Frankfurt, Frankfurt/Main, Germany; b Department of Biostatistics and Mathematical Models, Goethe University Frankfurt, Frankfurt/Main, Germany

**Keywords:** Hymenoptera venom allergy, Immunotherapy, Sting challenge, Quality of life

## Abstract

**Background:**

An experienced life-threating anaphylactic reaction to hymenoptera venom can sustainably impair patients’ quality of life (QoL). Besides carrying emergency medication, venom-specific immunotherapy (VIT) exists as a causal treatment of allergy.

**Objective:**

This study aimed to examine QoL, anxiety, depression, and physical and mental health in patients allergic to hymenoptera venom before and during VIT and the impact of a tolerated sting challenge (SC).

**Methods:**

Between July 2017 and August 2017, 142 patients with venom allergy were analyzed using validated questionnaires as the: Vespid Allergy Quality for Life Questionnaire" (VQLQ-d), the "Hospital Anxiety and Depression Scale" (HADS-D) and the "Short Form 36" (SF-36). To evaluate the impact of VIT and SC on the QoL, patients were divided into 3 groups: (A) VIT and tolerated SC (n = 45), (B) VIT before carrying out SC (n = 73), and (C) therapy-naïve before VIT (n = 20). Further parameters like gender, age, insect species, and severity of the anaphylactic reaction were assessed.

**Results:**

A significant correlation between the health-related QoL and the parameters of gender and state of treatment was seen. Especially male patients, as well as patients allergic to yellow jacket venom, benefit from a SC in terms of a significant increase in their QoL. In the total study cohort, a clear trend was observed towards a higher QoL in patients under VIT who tolerated a SC. Overall, neither the patients’ age nor the insect species exerted a relevant influence on QoL, depression or anxiety. However, women showed a lower QoL combined with higher anxiety and depression scores than men.

**Conclusion:**

Immunotherapy leads to an improved QoL, which can be further increased by a SC. A tolerated SC conceivably reassures the patients by objectifying the treatment success. Female patients appear to have a stronger impaired QoL per se. Taken together, a SC can be performed during VIT to strengthen the patients’ QoL.

## Introduction

Systemic allergic reactions to hymenoptera venom occur in 0.3–7.5% of adults, although large local reactions at the sting site are common in up to 26% of the population.^1^^,^^2^ Due to the potentially life-threating risk of an anaphylactic reaction, those affected by systemic allergic reactions can suffer from a severely impaired quality of life (QoL).^3^^,^^4^ Thus, both the patients’ physical health and mental well-being should be addressed by an adequate treatment.

One common emergency tool used to reduce the imminent lethal threat after a sting is epinephrine autoinjectors for self-administration. However, carrying these autoinjectors at all times is experienced as burdensome;^5^^,^^6^ more alarmingly, a considerable number of patients are insufficiently educated and equipped.^7^ In contrast, venom-specific immunotherapy (VIT) is a causal therapeutic approach that prevents the risk of a repeated allergic reaction in advance as well as provides an effective treatment in 77–84% of bee venom allergy patients and 91–96% of yellow jacket venom allergy patients.^8^, ^9^, ^10^ As reviewed by Dhami et al,^11^ VIT has been proven to be a safe procedure for both adults and children. In addition to its physiological therapeutic effectiveness, VIT is associated with a significant improvement in patients’ QoL.^5^^,^^6^^,^^12^, ^13^, ^14^, ^15^

The current European guidelines recommend VIT in adults and children with a systemic allergic reaction that exceeds generalized skin symptoms.^10^ However, VIT can be discussed as an option for adult patients with solely generalized cutaneous allergic symptoms (eg, urticaria, angioedema) who are either at high risk for re-exposure or who suffer from a severely impaired QoL.^10^^,^^14^

So far, the most reliable control for monitoring the clinical effectiveness of VIT is a sting challenge (SC).^10^ According to recommendations, it should be performed 6–18 months after the maintenance dose has been reached, in order to identify non-responders to VIT.^16^ In case of a non-response, presented by a systemic allergic reaction to the SC, an increase of the maintenance dose is advised.^10^ Risk factors for treatment failure include systemic reactions during VIT, allergy to bee venom, systemic mastocytosis, and elevated serum tryptase levels, as well as concomitant medication with inhibitors of the angiotensin-converting enzyme.^9^ One additional reason for recommending a SC under VIT is even greater improvement of the patient's QoL.^17^ However, a SC should neither be performed before VIT is started nor routinely after a completed course, in order to avoid boosting the allergy.^16^ Other absolute contraindications for a SC include severe and uncontrolled systemic diseases (eg, bronchial asthma, hypertension), pregnancy, and/or recurrent systemic reactions during the maintenance period just before the planned SC.^16^

This study's design is cross-sectional providing real-life data on the QoL of hymenoptera venom allergy patients undergoing VIT and a SC. Outcome parameters such as QoL, anxiety, depression, and physical and mental health were examined in 3 subgroups depending on their state of treatment: therapy-naïve patients before VIT, patients during VIT before a SC, and those under VIT after a tolerated SC. All patients were questioned over a short time period of 8 weeks in summer when the risk of a sting is relatively high and therefore impairment of the QoL more likely. While Fischer et al^17^ used a longitudinal matched design comparing patients before and after a SC, the aim of this study was to investigate the relevant benefit of a SC on QoL in a consistent and comparable setting for all patients at the time of the highest risk of exposure to hymenoptera.

## Patients and methods

### Patient data and cohort

In total, the cross-sectional study included 142 patients (57 men, 85 women) with a bee (n = 22) or yellow jacket (n = 120) venom allergy. All patients were studied in our allergology outpatient clinic in July and August 2017. Written informed consent was obtained from all patients. The study protocol was approved by the local Ethical Board (E49/17).

For further analysis, the patients were divided into 3 subgroups according to their state of treatment:

(A) during VIT and after a tolerated SC,
(B) during VIT before carrying out SC,
(C) therapy-naïve/before a planned VIT.

The severity of the anaphylactic reaction was graded according to the definition given by Ring and Meßmer.^18^

Four patients who failed the SC were defined as non-responders and were excluded from the study. Clinical characteristics of the patient cohort are shown in Table 1.

**Table 1:** Clinical characteristics

Patient cohort	
Total population (n)	142
Mean age (years)	52.21
Gender (n)	
Male	57
Female	85
Severity of reaction (n)	
°I	27
°II	71
°III	42
°IV	2
Insect venom allergy (n)	
bee	22
yellow jacket	120
State of treatment (n)	
Subgroup A	45 (20 male, 25 female)
Subgroup B	73 (25 male, 48 female)
Subgroup C	20 (12 male, 8 female)
Subgroup A (n)	
severity of reaction °I	4
severity of reaction °II	22
severity of reaction °III	18
severity of reaction °IV	1
yellow jacket venom allergy	38
bee venom allergy	7
Subgroup B (n)	
severity of reaction °I	15
severity of reaction °II	36
severity of reaction °III	21
severity of reaction °IV	1
yellow jacket venom allergy	66
bee venom allergy	7
Subgroup C (n)	
severity of reaction °I	8
severity of reaction °II	9
severity of reaction °III	3
severity of reaction °IV	–
yellow jacket venom allergy	16
bee venom allergy	4

### Outcome parameter

To assess the QoL, the German version of the Vespid Allergy Quality of Life Questionnaire (VQLQ-d) was performed.^19^ The VQLQ-d investigates emotional stress as well as impairments in daily routines, and it represents the gold standard for evaluation of the QoL in patients allergic to yellow jacket venom. Despite this condition, all patients of the presented cohort completed the VQLQ-d—that is, bee venom allergy sufferers were also evaluated with this questionnaire. The overall score falls in the range from 1 to 7, with 7 being the highest score representing an unrestricted QoL.

For further evaluation of anxiety and depression, the German version of the Hospital Anxiety and Depression Scale (HADS-D) was administered.^20^^,^^21^ An overall score of ≥11 in each of the categories is interpreted as positive for an existing anxiety or depression, whereas a score of ≤7 is interpreted as negative (neither anxiety nor depression existent). A score of 8–10 is defined as indifferent.

Another questionnaire targeting the health-related QoL is the Short Form 36 (SF-36), which consists of 8 domains: physical functioning, physical role, bodily pain, general health, vitality, social functioning, emotional role, and mental health.^22^^,^^23^ For this analysis, we condensed those domains into 2 main summary scores: physical health and mental health. The lower the score, the more impaired the patients’ QoL.

**Fig. 1:**
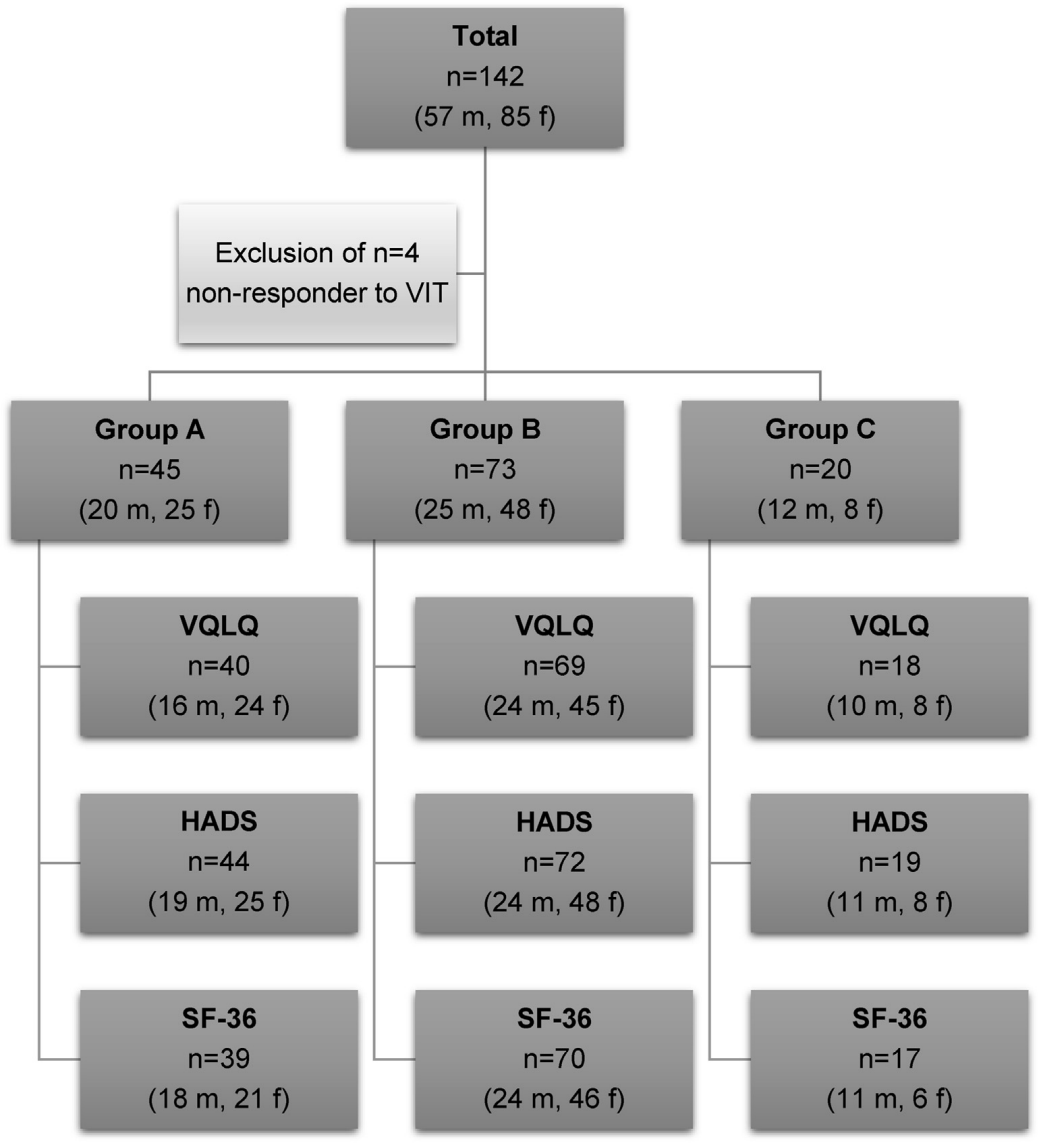
**Patient cohort**. This flowchart illustrates the sample sizes of the study population. The total patient cohort comprised n = 142 patients (57 men, 85 women). 4 female non-responders to VIT as well as any incorrectly completed questionnaire were excluded from statistical analysis. Thus, sample numbers can vary. The patients were divided into 3 subgroups according to their state of treatment: group A contains patients under VIT after a tolerated sting challenge, group B patients under VIT before carrying out sting challenge and group C therapy-naïve patients before VIT. Abbreviations: VIT = venom immunotherapy, m = male, f = female, VQLQ=Vespid Allergy Quality for Life Questionnaire, HADS=Hospital Anxiety and Depression Scale, SF-36 = Short Form 36

Only questionnaires that were correctly and fully completed were included in the final dataset. Therefore, sample numbers can vary (Fig. 1).

### Venom immunotherapy protocol

Depending on the presented allergy—bee or yellow jacket venom— venom-specific VIT was initiated after a detailed medical history was gathered, the grade of anaphylactic reaction was defined, and diagnostics including a skin prick test, venom-specific IgE levels, and serum tryptase were completed. The venom maintenance dose of 100 μg was achieved using a standardized 2-day ultra-rush protocol with the purified preparations ALK-lyophilized bee venom SQ® 801 and ALK-lyophilized vespid venom SQ® 802 (ALK-Abelló, Denmark) and was administered in an inpatient setting. Subsequently, we continued with a maintenance dose of 100 μg at 4–6 week intervals in our allergology outpatient clinic using aluminum hydroxide-absorbed venom extracts (ALK-depot SQ insect-venom). After 6–18 months, a SC was performed in an inpatient setting according to the protocol in the EAACI position paper^9^^,^^24^ under anesthesiological surveillance. If the patient tolerated the SC, the maintenance dose was continued for the total course of treatment (5 years). The precondition for participation in the SC was the patient's agreement to continue VIT for at least another 6 months.

### Statistics

Statistical analysis of the resultant parameters was performed with the program “BiAS” (Version 11.10, epsilon-Verlag 1989–2020, Frankfurt, Germany). Normal distribution was tested with the Kolmogorov-Smirnov test. Due to non-normally distributed outcome parameters, Wilcoxon rank-sum test and Kruskal-Wallis (KW) test for comparison of independent samples were applied. For post-hoc analyses, Conover-Iman test and Holm-Bonferroni method were performed. Additionally, effect sizes according to Rosenthal (R) and Rasch (eta^2^) were calculated. As for the correlation analysis, Spearman correlation was conducted. Thus, all results are displayed as median values and minimum-to-maximum ranges. The level of statistical significance was set at *p* < 0.05.

## Results

### Quality of life

**Fig. 2:**
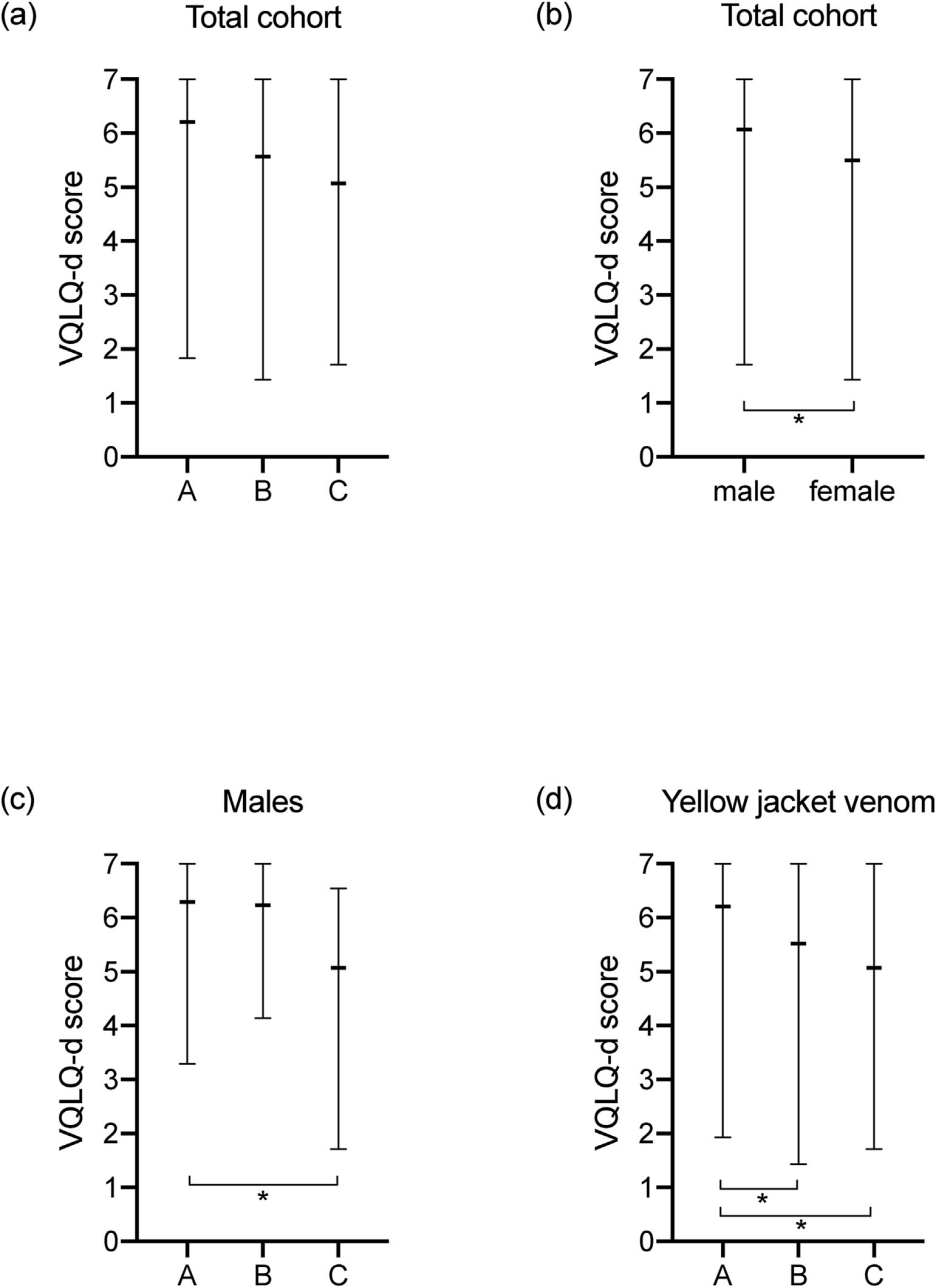
**Subgroup analysis for quality of life**. This figure illustrates the quality of life (QoL) measured by the VQLQ-d according to the state of treatment. Patients were divided into 3 subgroups: (A) VIT after a tolerated sting challenge, (B) VIT before carrying out a sting challenge and (C) therapy-naïve patients before VIT. Panel (a) shows the overall cohort stratified by treatment subgroups: post-hoc analyses did not detect significant between-group differences. Panel (b) plots the gender-specific differences in the overall cohort, where women exhibited a significantly more impaired QoL than men (*p* = 0.0347 using Wilcoxon rank-sum test). In panel (c) exclusively male patients are stratified by treatment subgroups: group A showed a significantly higher QoL than group C (*p* = 0.0270 in post-hoc analyses). Panel (d) depicts solely patients allergic to yellow jacket venom: group A displayed a significantly increased QoL compared to group B and C (*p* = 0.0344 respectively in post-hoc analyses). For comparison of 3 samples Kruskal-Wallis test and for post-hoc analyses Conover-Iman test and Holm-Bonferroni method were applied. The level of statistical significance was set at *p* < 0.05

A total of 127 completed VQLQ-d forms (50 men, 77 women) were included in the statistical analysis. Overall, a clear trend towards an increased QoL in patients under VIT who had tolerated the SC (A) compared to patients under VIT who had not yet undergone a SC (B) or therapy-naïve patients (C) was observed (KW test: *p* = 0.0285). However, in post-hoc analyses no significant between-group differences were identified (Table 2). In terms of gender differences, female patients showed overall a significantly more impaired QoL than male patients (Table 2). Fig. 2 illustrates these VQLQ-d results according to treatment subgroup and to gender.

**Table 2:** Quality of life according to VQLQ-d. Significant differences are highlighted in bold.

	n	median (min/max)	*p*-value (KW test)	*p*-value (post-hoc test)	effect size
QoL (A)	40	6.21 (1.83/7.00)	**0.0285**	A vs. B: 0.0599	eta^2^ = 0.0565
QoL (B)	69	5.57 (1.43/7.00)		A vs. C: 0.0510	
QoL (C)	18	5.07 (1.71/7.00)		B vs. C: 0.3460	
QoL Male	50	6.07 (1.71/7.00)	**0.0347**		R = 0.1874
QoL Female	77	5.50 (1.43/7.00)			
QoL Male (A)	16	6.29 (3.29/7.00)	0.1802		R = 0.2119
QoL Female (A)	24	5.99 (1.83/7.00)			
QoL Male (B)	24	6.23 (4.14/7.00)	**0.0070**		R = 0.3246
QoL Female (B)	45	5.36 (1.43/6.69)			
QoL Male (C)	10	5.07 (1.71/6.54)	0.6965		R = 0.1152
QoL Female (C)	8	5.40 (2.57/7.00)			
QoL Male (A)	16	6.29 (3.29/7.00)	**0.0331**	A vs. B: 0.3474	eta^2^ = 0.1391
QoL Male (B)	24	6.23 (4.14/7.00)		A vs. C: **0.0270**	
QoL Male (C)	10	5.07 (1.71/6.54)		B vs. C: 0.0815	
QoL Female (A)	24	5.99 (1.83/7.00)	0.1652		eta^2^ = 0.0474
QoL Female (B)	45	5.36 (1.43/6.69)			
QoL Female (C)	8	5.40 (2.57/7.00)			
QoL (°I)	26	6.04 (3.36/6.93)	**0.0291**	°I vs. II: 0.0755	eta^2^ = 0.0561
QoL (°II)	63	5.36 (1.71/7.00)		°I vs. III-IV: 0.8803	
QoL (°III-IV)	38	6.22 (1.43/7.00)		°II vs. III-IV: 0.0755	
QoL (°I, A)	3	6.93 (5.50/6.93)	0.1748		eta^2^ = 0.0894
QoL (°II, A)	20	5.70 (1.83/6.86)			
QoL (°III-IV, A)	17	6.31 (1.93/7.00)			
QoL (°I, B)	15	6.08 (3.46/6.62)	0.0929		eta^2^ = 0.0699
QoL (°II, B)	35	5.36 (2.36/6.69)			
QoL (°III-IV, B)	19	6.14 (1.43/7.00)			
QoL (°I, B)	8	5.89 (3.36/6.57)	0.2199		eta^2^ = 0.1860
QoL (°II, B)	8	4.90 (1.71/7.00)			
QoL (°III-IV, B)	2	4.53 (3.92/5.14)			
QoL (bee, A)	5	5.77 (1.83/6.93)	0.9931		eta^2^ = 0.0010
QoL (bee, B)	7	6.29 (1.45/6.62)			
QoL (bee, C)	4	5.18 (3.86/6.57)			
QoL (yj, A)	35	6.21 (1.93/7.00)	**0.0138**	A vs. B: **0.0344**	eta^2^ = 0.0779
QoL (yj, B)	62	5.52 (1.43/7.00)		A vs. C: **0.0344**	
QoL (yj, C)	14	5.07 (1.71/7.00)		B vs. C: 0.3481	

Abbreviations: QoL = quality of life, yj = yellow jacket. Subgroup analysis: (A) patients under VIT after a tolerated sting challenge, (B) patients during VIT before carrying out sting challenge, (C) therapy-naïve patients before VIT, °I-IV severity of anaphylactic reaction according to the definition of Ring and Messmer^18^. Wilcoxon rank-sum test and Kruskal-Wallis (KW) test with Conover-Iman test and Holm-Bonferroni method for post-hoc analyses were performed. The level of statistical significance was set at *p* < 0.05. Effect sizes were calculated according to Rosenthal (R = 0.1 small, R = 0.3 medium, R = 0.5 large, R > 0.7 very large effect) and Rasch (eta^2^ = 0.01 small, eta^2^ = 0.06 medium, eta^2^ = 0.14 large effect)

Considering exclusively men, a significantly higher QoL was detected in group A compared to C (Table 2). In contrast, no significant differences were found within the female cohort between group A, B, or C. Likewise, in the subset of patients allergic to yellow jacket venom group A displayed a significantly higher QoL than group B or C (Table 2). However, these effects were not observed in patients allergic to bee venom.

Patients with a grade III or IV reaction showed a higher QoL compared to a grade I or II reaction, as the latter showed a lower QoL. However, these differences were not significant in post-hoc analyses (Table 2). Furthermore, it should be noted that due to small sample sizes, patients with a grade III or IV reaction were combined into one group.

Ultimately, in the overall cohort neither the patients’ age nor the type of insect allergy exerted a significant influence on QoL.

### Anxiety and depression

The overall number of completed HADS-D questionnaires was 135 (54 men, 81 women). Positive scores (≥11) were seen in 13 patients (9.6%) for the anxiety domain and in 8 patients (5.9%) for the depression domain. An indifferent score (8–10) was seen in 11 patients (8.2%) for anxiety and in 8 patients (5.9%) for depression. A negative score (≤7) was seen for 111 patients (82.2%) for anxiety and in 119 patients (88.2%) for depression.

Overall, women showed significantly higher anxiety and depression scores than men (Table 3). No significant correlation was detected between the state of treatment and the level of anxiety; however, patients with a grade I reaction displayed significantly lower scores for depression than patients with a grade II reaction (Table 3).

**Table 3:** Anxiety and depression according to HADS-D. Significant differences are highlighted in bold.

	n	median (min/max)	*p*-value (KW test)	*p*-value (post-hoc tests)	effect size
Anxiety (A)	44	4.5 (0/18)	0.3090		eta^2^ = 0.0175
Anxiety (B)	72	5.0 (0/16)			
Anxiety (C)	19	2.0 (0/15)			
Depression (A)	44	2.0 (0/18)	0.9862		eta^2^ = 0.0002
Depression (B)	72	1.0 (0/15)			
Depression (C)	19	1.0 (0/14)			
Anxiety Male	54	3.0 (0/14)	**0.0017**		R = 0.2697
Anxiety Female	81	5.0 (0/18)			
Anxiety Male (A)	19	3.0 (0/10)	0.1366		R = 0.2302
Anxiety Female (A)	25	5.0 (0/18)			
Anxiety Male (B)	24	4.0 (0/11)	0.2412		R = 0.1401
Anxiety Female (B)	48	5.0 (0/16)			
Anxiety Male (C)	11	1.0 (0/14)	**0.0121**		R = 0.5791
Anxiety Female (C)	8	6.0 (1/15)			
Depression Male	54	1.0 (0/14)	**0.0238**		R = 0.1945
Depression Female	81	2.0 (0/18)			
Depression Male (A)	19	1.0 (0/14)	0.0575		R = 0.2938
Depression Female (A)	25	3.0 (0/18)			
Depression Male (B)	24	1.0 (0/13)	0.5649		R = 0.0704
Depression Female (B)	48	1.5 (0/15)			
Depression Male (C)	11	1.0 (0/4)	**0.0409**		R = 0.4979
Depression Female (C)	8	3.5 (0/14)			
Anxiety (°I)	27	4.0 (0/11)	0.1019		eta^2^ = 0.0341
Anxiety (°II)	67	5.0 (0/18)			
Anxiety (°III-IV)	41	4.0 (0/16)			
Depression (°I)	27	1.0 (0/13)	**0.0058**	°I vs. II: **0.0037**	eta^2^ = 0.0768
Depression (°II)	68	2.0 (0/18)		°I vs. III-IV: 0.1086	
Depression (°III-IV)	40	1.5 (0/15)		°II vs. III-IV: 0.1817	

Subgroup analysis: (A) patients under VIT after a tolerated sting challenge, (B) patients during VIT before carrying out sting challenge, (C) therapy-naïve patients before VIT, °I-IV severity of anaphylactic reaction according to the definition of Ring and Messmer^18^. Wilcoxon rank-sum test and Kruskal-Wallis (KW) test with Conover-Iman test and Holm-Bonferroni method for post-hoc analyses were performed. The level of statistical significance was set at *p* < 0.05. Effect sizes were calculated according to Rosenthal (R = 0.1 small, R = 0.3 medium, R = 0.5 large, R > 0.7 very large effect) and Rasch (eta^2^ = 0.01 small, eta^2^ = 0.06 medium, eta^2^ = 0.14 large effect)

Again, no influence was found due to the patients’ age or the type of insect allergy.

### Physical and mental health

A total of 126 patients (53 men, 73 women) completed the SF-36 questionnaire. The medians for the physical health summary score were 54.2, 53.0, and 55.7 for subgroups A, B, and C respectively; for the mental health summary score, the medians were 54.6 (A), 54.3 (B), and 54.6 (C).

For the overall cohort, no significant correlation was found between the SF-36 results and the following parameters: state of treatment, gender, severity of anaphylactic reaction, or type of insect allergy.

In the total study population as well as in subgroup B, the patients’ age correlated negatively with the physical health score, meaning that older patients displayed lower physical health summary scores. Interestingly, for mental health and age, a positive correlation was found only in subgroup B (patients under VIT who had not yet undergone a SC) (Table 4). In other words, those patients presented higher mental health summary scores with increasing age.

**Table 4:** Influence of age on the physical and mental health. Significant differences are highlighted in bold.

	n	Spearman's correlation coefficient (rho)	Edgeworth approximation (*p*-value)
**Physical health**			
Subgroup (A)	39	−0.1061	0.5191
Subgroup (B)	70	−0.3762	**0.0014**
Subgroup (C)	17	−0.3620	0.1529
**Mental health**			
Subgroup (A)	39	−0.0202	0.9027
Subgroup (B)	70	0.2800	**0.0192**
Subgroup (C)	17	−0.0773	0.7656

Correlation analysis between the variable age and both SF-36 summary scores (physical and mental health) using Spearman's correlation coefficient (rho). Rho was rated according to Evans as follows: <0.2 poor, 0.2–0.4 weak, 0.4–0.6 moderate, 0.6–0.8 strong, >0.8 optimal correlation. A negative sign represents a reverse correlation. The level of statistical significance was set at *p* < 0.05 using Edgeworth approximation. Subgroups: (A) patients under VIT after a tolerated sting challenge, (B) patients during VIT before carrying out sting challenge, (C) therapy-naïve patients before VIT

## Discussion

Experiencing an anaphylactic reaction after an insect sting can have broad consequences for patients, not only as a life-threatening event per se but also by having a lasting influence on their QoL.^3^ Multiple studies demonstrated that VIT has a substantially beneficial effect on QoL in patients with a hymenoptera venom allergy.^4^, ^5^, ^6^^,^^12^, ^13^, ^14^, ^15^^,^^17^^,^^25^^,^^26^ In this study we applied an ultra-rush protocol for initiating VIT, which has been proven as a safe and fast option for the build-up phase.^27^ A controlled SC is the gold standard for identifying non-responder to VIT. When carried out under medical supervision it provides a valid verification of therapeutic effectiveness, while having the advantage of a more controlled setting than a field sting.^28^ Thus, the relevance of inpatient allergology is vital for patients with hymenoptera venom allergy.

The study's patient population is a cross-section of patients with hymenoptera venom allergy treated in our allergological outpatient clinic. All patients were questioned over a short time period during summer time when the risk of a sting is relatively high and therefore impairment of the QoL more likely.

Cohort stringency concerning age, gender, and insect allergy is provided compared to other cohorts.^25^^,^^29^ Whereas in the present population the number of female patients slightly prevails, epidemiologic data show a higher prevalence of sensitization to hymenoptera venom in males.^30^ The rate of patients allergic to either bee or yellow jacket venom can vary depending on the degree of exposure. It should be noted that in the present study cohort the sample size of patients allergic to bee venom was rather small. Therefore, this group is underrepresented and the statistical as well as clinical significance of subgroup analyses in this subset of patients is limited.

The present study reveals differences in QoL in patients allergic to bee or yellow jacket venom in relation to their state of treatment. Patients under VIT who had tolerated a SC displayed the highest QoL, and those under VIT who had not yet performed a SC demonstrated a higher QoL than therapy-naïve patients. This clear trend failed to reach statistical significance in post-hoc tests, possibly due to rather small sample sizes. However, subgroup analyses revealed that particularly men and patients allergic to yellow jacket venom responded to a tolerated SC with an increased QoL. By contrast, this effect was not found in bee venom allergy sufferers; thus, their QoL seems less influenced by the state of treatment. Amongst these are often beekeepers who tend to have a less anxious relationship to their insects. Interestingly, one German study on bee venom allergy in beekeepers showed that only half of the allergic beekeepers wore full protective clothing.^31^

While Nowak et al^13^ found a significantly poorer QoL in patients allergic to wasp venom, Koschel et al^25^ showed a more pronounced improvement of the QoL in patients allergic to yellow jacket venom after SC, respectively, compared to patients allergic to bee venom. However, we did not detect an influence of the type of insect allergy on the QoL in our overall cohort. Regardless, a SC should be performed in patients allergic to bee venom due to their higher risk of possible treatment failure to VIT.^8^, ^9^, ^10^ It should be noted that all patients completed the VQLQ-d, independent of their insect-specific allergy. Even if this questionnaire has only been validated for patients allergic to yellow jackets, it is common practice to administer it to patients allergic to bee venom.^13^^,^^25^^,^^32^ Additionally, the discrimination rate between hymenoptera types in the general population is rather poor.^33^ The favorable effect of a SC within a VIT regime on patient QoL was already demonstrated by others.^17^^,^^25^ An enhanced QoL in patients under VIT was independent of the patients’ age, as found in the existing literature.^4^^,^^25^

Patients with a grade III or IV anaphylactic reaction displayed the highest VQLQ-d scores, while those with a grade II reaction displayed the most impaired QoL. Although this was not significant in post-hoc analyses, it suggests that the “sickest” patients do not necessarily experience the worst QoL and highlights the individualized character of QoL in patients with hymenoptera allergy. Several other previous studies did not find a correlation between severity of reaction and health-related QoL.^4^^,^^13^^,^^25^

Gender appears to play a crucial role in evaluations of the QoL. Our results indicate that female patients displayed a more impaired QoL and showed higher levels of anxiety and depression compared to male patients. These findings match the results of several previously-published studies,^4^^,^^5^^,^^15^^,^^17^ although other studies found gender to be uninfluential.^25^ One study examining HADS values in the general German population reported that women were more anxious than men, although depression levels were comparable.^34^ The disposition towards an increased rate of anxiety and depression in female patients might explain why a beneficial effect of VIT and a SC on their QoL remains concealed.

No correlation was found between the severity of anaphylactic reaction and anxiety, whereas patients with grade I reactions showed a significantly lower level of depression compared to patients reacting grade II. Otherwise, patients with a severe reaction (III and IV) did not seem more anxious or depressed than patients with a grade I or II reaction. However, the distinct and rather small sample sizes might be a possible confounder, and we suggest a reevaluation with a bigger cohort. As for anxiety, ambiguous findings exist in terms of its correlation with severity of the anaphylactic reaction: while Schaarschmidt et al^29^ did not find an association between these parameters, Cichocka-Jarosz et al^32^ revealed a significant correlation in their cohort. Still, we conclude that treatment indications for VIT should not only be based on the severity of the reaction, but should also include an assessment of the QoL. In fact, even anxious patients with a systemic reaction that is limited to cutaneous symptoms but with a distinctly impaired QoL can benefit from VIT.^14^

No significant correlation was detected between state of treatment and depression or anxiety scores. Schaarschmidt et al^29^ showed that neither the status of VIT nor the duration of VIT had an influence on anxiety in their cohort, while Nowak et al^13^ demonstrated a decreased intensity of anxiety under VIT. Interestingly, one study^25^ reported a post-SC increase in depression, measured by the General Severity Index of the Brief Symptom Inventory. The authors argued that depression was possibly masked by anxiety before the SC.^25^ Apart from this, debilitating beliefs and emotional distress can persist after VIT independent of age, sex, or education, and exerting a long-lasting influence on perceived QoL.^3^

In the present study's cohort, only 9.6% of the patients showed positive HADS-D test scores (≥11) for anxiety, and 5.9% of all patients displayed positive scores for depression. In comparison, Findeis et al^26^ reported non-normal depression values in 20% of their patients (as measured by the Hamilton Depression Index); Schaarschmidt et al^29^ claimed significant values in 5.5% of their cohort for depression and 14.5% for anxiety; and Nowak et al^13^ found a rate of approximately 12% for each disorder. In the general German population, Hinz et al^34^ found elevated anxiety and depression levels of 21% and 23%, respectively, when administering the HADS and using 8+ as a cut-off value (compared to 17.8% for anxiety and 11.8% for depression in our study population, with 8+ as the cut-off value). Therefore, it seems that patients who are allergic to hymenoptera venom are not more anxious or depressed per se.

Overall, our patient cohort seems rather healthy regarding psychological comorbidities, with approximately 82–88% of the patients displaying HADS-D values within the normal range and thus having little room for improvement under treatment. Nevertheless, patients with impaired mental health should be identified beforehand for closer monitoring under therapy and, if necessary, should be provided with additional support.

In terms of physical and mental health—as measured by the SF-36—no significant correlation was found for the outcome variables of state of treatment, gender, severity of anaphylactic reaction, or type of insect allergy. Koschel et al^25^ did not find a significantly altered physical or mental SF-36 score after a SC was performed. Our data indicated a negative or reverse correlation between patients’ age and physical health; in other words, older patients felt less functional. This can be attributed to the natural course of aging. Decline of the physical component summary score of the SF-36 with increasing age has also been shown by other studies.^35^^,^^36^ As for mental health, a positive correlation with age was only found in patients under VIT without having undergone a SC yet, suggesting a more self-effective attitude with increasing age in this subset of patients. However, the significance of this finding is limited, since this correlation was only seen in one of the subgroups. A trend towards increase of the mental component summary score until the age of 69 has been described elsewhere.^35^^,^^36^

## Conclusion

This study provides real-life data on QoL in patients with hymenoptera venom allergy in relation to their state of therapy. The cross-sectional study design offers a comparable setting by reducing possible bias such as an altered risk of exposition to the insect due to seasonal changes. Even if the present study cohort is representative, we recommend a validation in a bigger patient cohort in a preferable multicentric setting.

Our study emphasizes the importance of assessing QoL in patients with a hymenoptera venom allergy and confirms a difference in relation to their state of treatment. While for the overall cohort a clear trend towards an increased QoL in patients under VIT and further after tolerating a SC was observed, subgroup analyses revealed that especially men and patients allergic to yellow jacket venom benefit from a SC with a significantly higher QoL. We assume that a tolerated SC reassures the affected patients of their self-efficacy by objectifying the treatment's success. Female patients appear to perceive their QoL as more impaired than male patients, and they show higher levels of anxiety and depression. To ensure optimal care for patients in everyday clinical practice we recommend a supplemental assessment of QoL. Beyond that, a SC can be performed during VIT to strengthen patients' QoL and induce an extra benefit.

## Abbreviations

HADS-D: Hospital Anxiety and Depression Scale; KW: Kruskal-Wallis; QoL: quality of life; SC: sting challenge; SF-36: Short Form 36; VIT: venom-specific immunotherapy; VQLQ-d: Vespid Allergy Quality of Life Questionnaire

## Funding

This research did not receive any specific grant from funding agencies in public, commercial, or not-for-profit sectors.

## Availability of data and materials

All relevant data are within the paper.

## Author contributions

All the signing authors contributed to the clinical work (EV, KA, MM, RK, SK), data collection (TE, KNZ, EV, KA) and analysis of the data and drafting the manuscript (TE, EV, HA, KNZ, MM, RK, NZ). EV and HA designed the study. HA was the biostatistician.

## Ethics approval

Study protocol was approved for data collection by the ethic commission, Goethe-University of Frankfurt, Germany (No. 96/17). The described treatments were part of the standard of care in this field.

## Consent for publication

All authors approved the final version and its submission.

## Declaration of competing interest

TE, KNZ, KA, HA, NZ, MM, RK and SK certify, that they have NO affiliations with or involvement in any organization or entity with any financial interest or non-financial interest in the subject matter or materials discussed in this manuscript. EV declares intermittent advisory board relationship with ALK-Abelló. The authors certify, that there were NO funding sources supporting the work.
